# The Conversion of Wolff-Parkinson-White (WPW) Pattern into WPW Syndrome in the Presence of Ischemia: A Case Report

**DOI:** 10.7759/cureus.4147

**Published:** 2019-02-27

**Authors:** Arshad Muhammad Iqbal, Muhammad Salman Ghazni, Ateeq Mubarik, Nida Zubair, Syed Farrukh Jamal

**Affiliations:** 1 Internal Medicine, Oak Hill Hospital, Brooksville, USA; 2 Cardiology, Tabba Heart Institute, Karachi, PAK; 3 Internal Medicine, Dow Medical College and Civil Hospital Karachi, Karachi, PAK; 4 Cardiology, National Institute of Cardiovascular Diseases (NICVD), Karachi, PAK

**Keywords:** wolff-parkinson-white (wpw) pattern, wpw syndrome, acute myocardial infarction (ami), electrocardiogram (ekg)

## Abstract

Wolff-Parkinson-White (WPW) syndrome is defined by the presence of a short PR interval, delta waves on the electrocardiogram (EKG), and symptomatic tachycardia. The condition is rare but can be life-threatening if not recognized in a timely manner. The WPW pattern on EKG can mask ischemic changes and may also increase the risk of arrhythmia and subsequent mortality. Our case describes the conversion of an underlying WPW pattern into WPW syndrome in the scenario of an acute myocardial infarction (AMI).

## Introduction

The prevalence of a Wolff-Parkinson-White (WPW) pattern on the surface electrocardiogram (EKG) is rare and estimated at 0.13% to 0.25% in the general population [1-2]. In 1930, it was initially described by Wolff, Parkinson, and White [3]. The diagnosis of acute myocardial infarction can be difficult on EKG when there are underlying abnormalities due to WPW. Case reports have been published in the past describing the coexistence of WPW syndrome and myocardial infarction (MI) [4]. We report a similar case where ischemia supposedly unmasked the syndrome, which was previously labeled as a WPW pattern with no previous clinical sequelae.

## Case presentation

**Figure 1:**
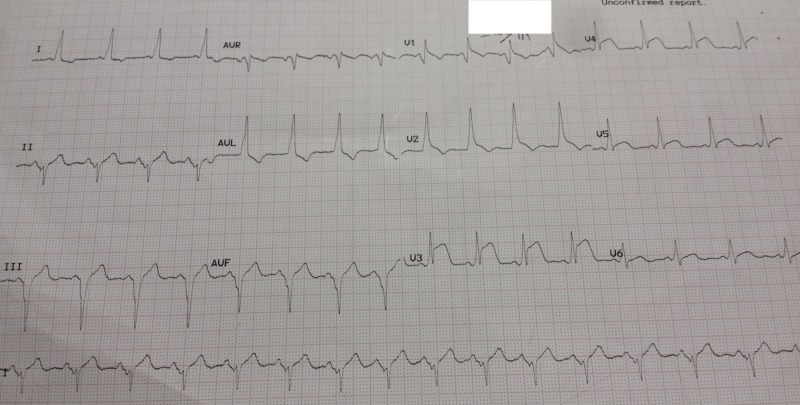
Electrocardiogram on arrival showing ST-segment elevation (V3 - V5), delta waves, short PR interval, and reciprocal ST-segment depression (I, aVL).

A 45-year-old male with a history of a Wolff-Parkinson-White pattern was admitted to the hospital with complaints of a sudden onset of chest heaviness radiating to left arm, along with profuse sweating, for two hours. His family history was unremarkable. He denied smoking, alcohol, cocaine, and tobacco use. Physical examination revealed a blood pressure of 160/100 mmHg and a regular pulse rate of 100/min. The remainder of the physical examination was unremarkable. EKG on arrival revealed sinus rhythm with a right bundle branch block (RBBB), normal axis, and ST-segment elevation of 2 mm in leads V3-V5 with reciprocal changes in leads I and aVL. A short PR interval with delta waves was also observed in the EKG (Figure 1). In light of the patient's symptoms and EKG findings, a diagnosis of acute myocardial infarction (AMI) with a WPW pattern was made. Immediate therapy, including aspirin, clopidogrel, metoprolol, nitroglycerine, and heparin, was administered, and the patient was rushed for an emergent coronary angiogram. 

**Figure 2:**
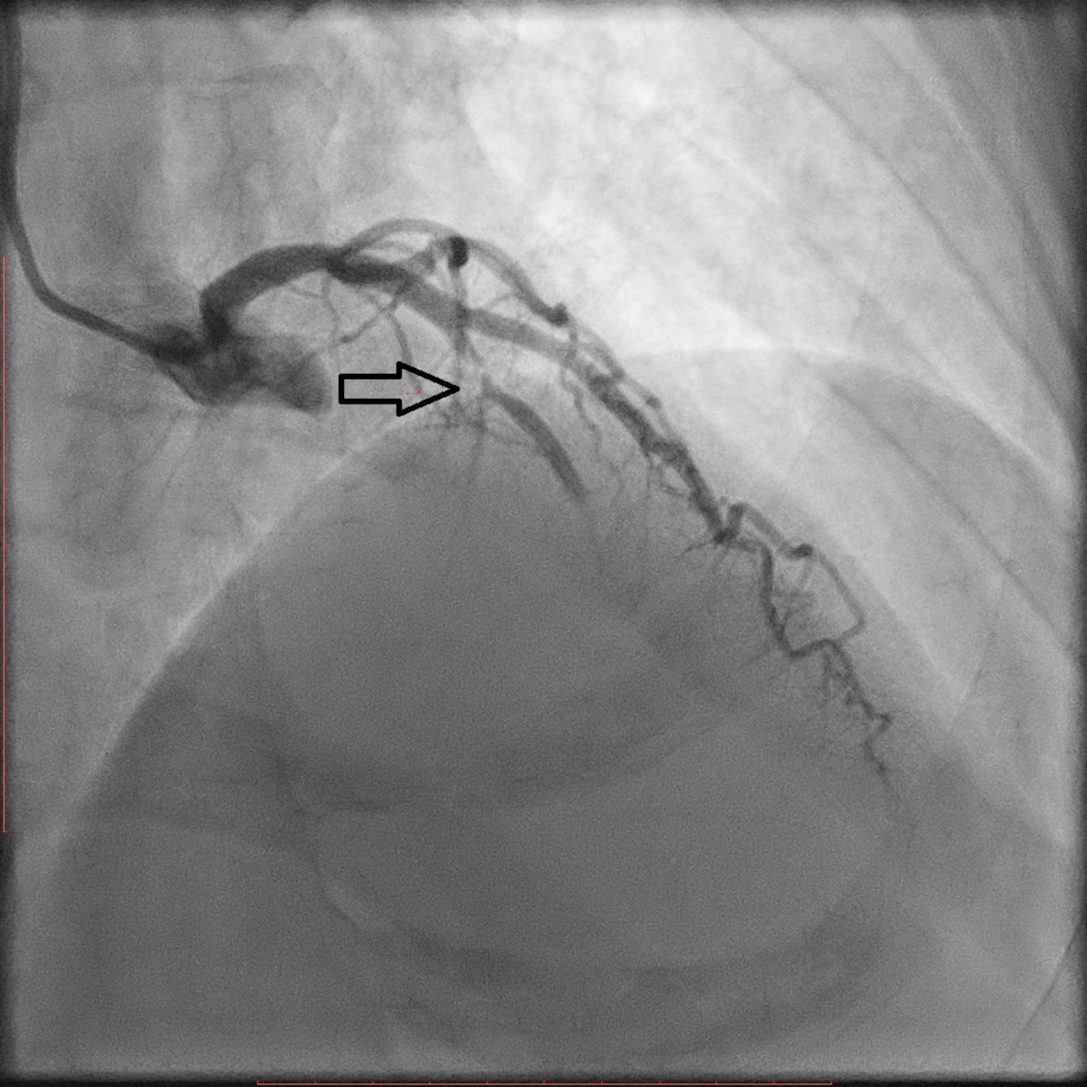
Coronary angiogram showing a severe obstructive lesion (arrowhead) in the mid-left anterior descending (LAD) artery.

**Figure 3:**
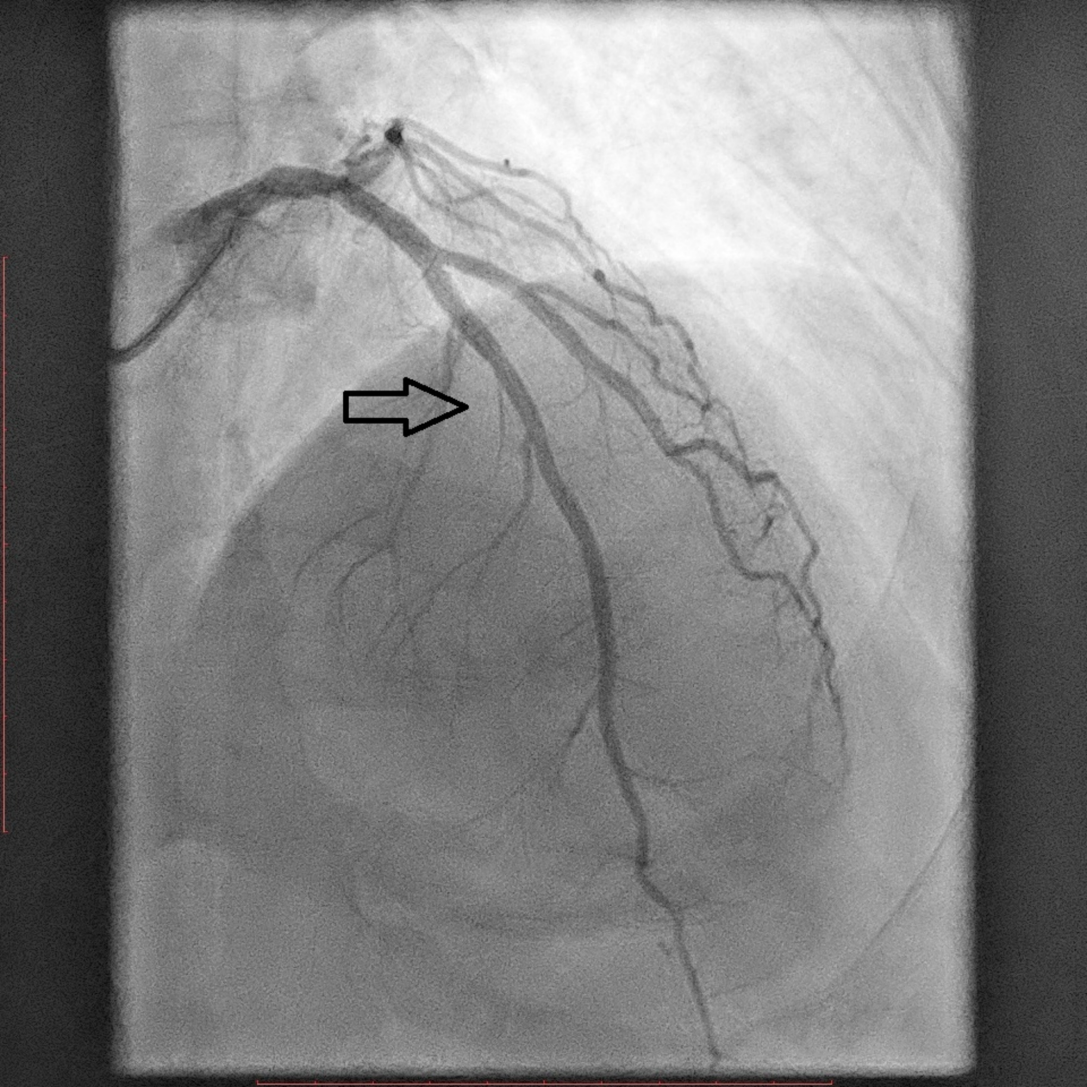
Coronary angiogram showing the deployment of a mid-left anterior descending (LAD) stent (arrow).

**Figure 4:**
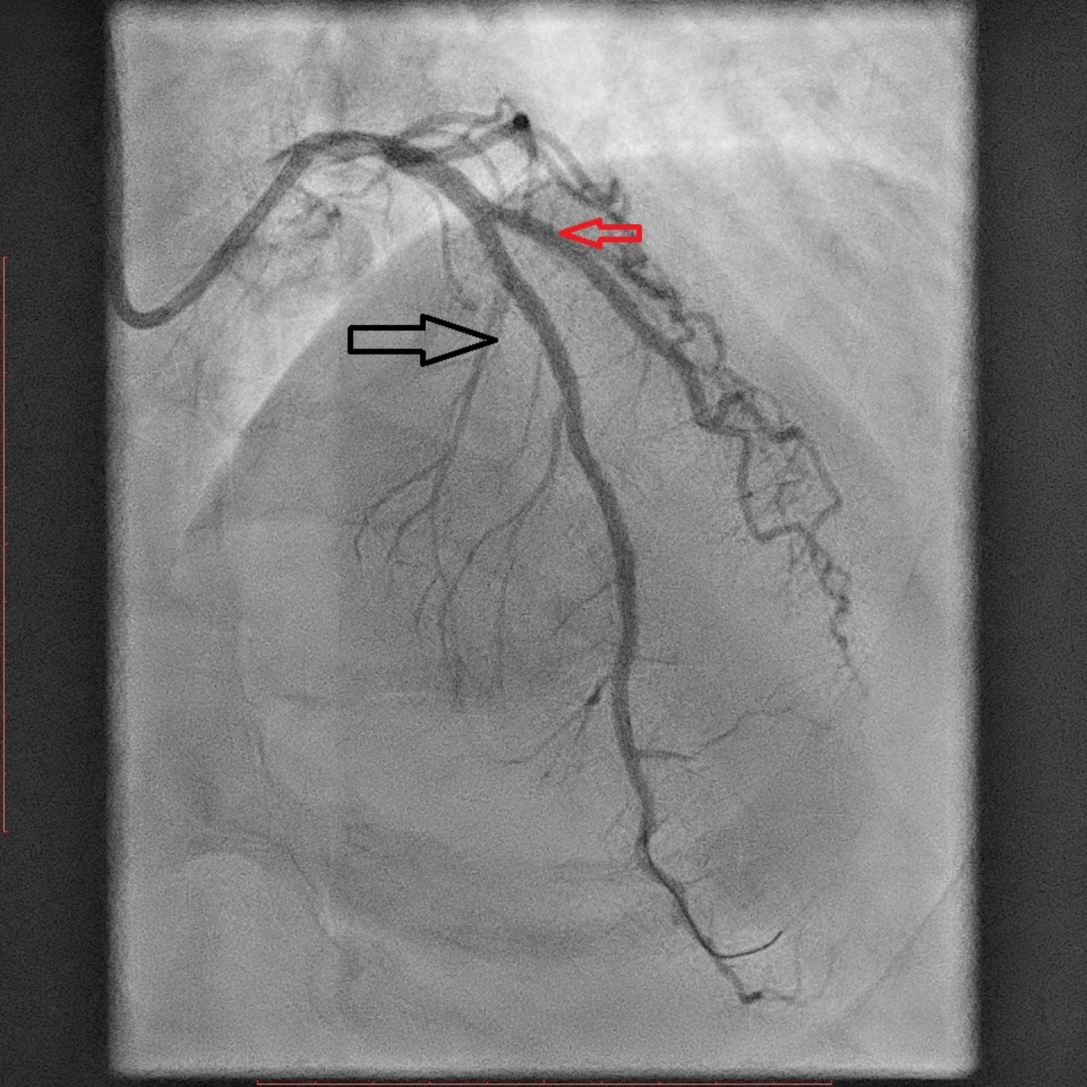
Post-PCI, TIMI-3 flow was achieved (black arrow). Normal blood flow to the diagonal branch is also shown by the red arrow. PCI: percutaneous coronary intervention; TIMI: thrombolysis in myocardial infarction

**Figure 5:**
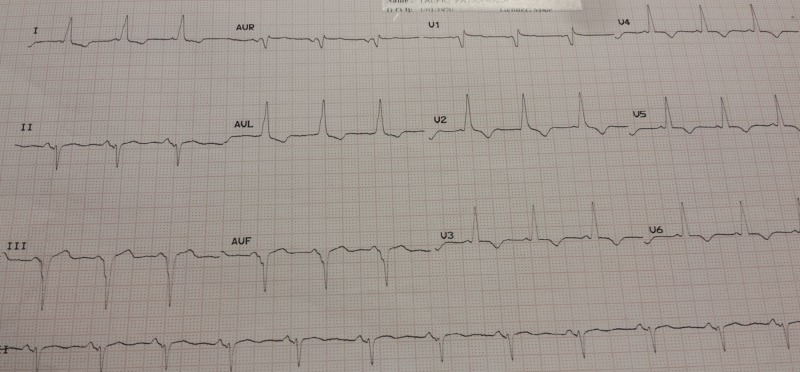
Electrocardiogram after primary PCI with stenting showing complete resolution of the ST segment changes PCI: percutaneous coronary intervention

A coronary angiogram revealed a nondominant right coronary artery (RCA), a non-obstructive left circumflex (LCX) artery, and a severe lesion in the mid-segment of the left anterior descending (LAD) artery (Figure 2). Primary percutaneous coronary intervention (PPCI) to the LAD was performed, and a drug-eluting stent (DES) was deployed (Figure 3). During the procedure, the patient developed narrow complex regular tachycardia with poor hemodynamics, so synchronized electrical cardioversion was performed with 100 joules. Post-percutaneous coronary intervention (PCI), thrombolysis in myocardial infarction (TIMI)-3 flow was achieved successfully (Figure 4). Complete resolution of the patient's symptoms and EKG changes were reported after the angioplasty (Figure 5). 

The laboratory evaluation, including a complete blood count (CBC), serum creatinine (Cr), glycated hemoglobin (HbA1c), and posterior-anterior chest X-ray, was within normal limits pre- and post-procedure. Cardiac troponin-I levels came back elevated with a serially rising trend (0.3 ng/ml, 28 ng/ml, 37ng/ml). The echocardiogram revealed moderate left ventricular systolic dysfunction with apical and septal akinesia and an ejection fraction (EF) of 35% with Grade I left ventricular (LV) diastolic dysfunction. The rest of the hospital course was unremarkable, and the patient was discharged home in stable condition. Follow-up echocardiogram at six weeks revealed improvement in the LV systolic function (left ventricular ejection fraction (LVEF) was 45% with apical akinesia). The patient was followed up for two years after the event, and at the last recent follow-up, there were no reported symptoms or events. 

## Discussion

The WPW pattern is applied to the patient with pre-excitation manifest on an EKG in the absence of symptomatic arrhythmias. The WPW syndrome is applied to the patient with both pre-excitation manifest on an EKG and symptomatic arrhythmias involving the accessory pathway. The prevalence of a WPW pattern on the surface EKG is estimated at 0.13% to 0.25% in the general population [5]. The WPW pattern on the EKG may be intermittent and may even disappear over time [6]. In one study, 22% of individuals who eventually manifest the WPW pattern on an EKG initially had a normal tracing, and in 40% of these individuals, the WPW pattern subsequently disappeared [2].

The WPW syndrome is amongst the most common pre-excitation syndromes. It was estimated to occur in approximately 0.1% to 3% of the general population [7]. Most of them (60% to 70%) had no pre-existing cardiac disease. Males dominated females with a 3:2 ratio [8]. Characteristic features were defined as:

• Short PR interval < 120 milliseconds (ms)

• Pre-excited depolarization of ventricles causing slurring of the initial QRS complex (delta wave)

• Widening of QRS complex > 110 ms

• PJ interval (beginning of P to end of QRS complex) < 260 ms

• Secondary repolarization changes (ST segment and T wave deflection inverse to the major QRS complex deflection)

Although the majority of patients with pre-excitation remain asymptomatic throughout their lives, sudden death has been reported with ventricular fibrillation being the usual mechanism [9]. EKG of patients with pre-excitation may mimic other conditions, including myocardial infarction, ventricular bigeminy, accelerated idioventricular rhythm, or electrical alternans [9-10]. In 1976, Ruskin et al. reported that among the 44 patients with WPW syndrome referred to their institution, 70% had EKG findings simulating a Q wave myocardial infarct pattern [11]. Guler et al. described a patient who was successfully resuscitated from ventricular fibrillation and whose initial EKG showed ST-segment elevation in precordial leads, suggesting an acute anterior myocardial infarction [9]. Poh et al. also described a similar case of WPW but with early repolarization changes in the EKG which could mimic myocardial infarction [12].

The prompt recognition and timely reperfusion are not only therapeutic goals but also considered the quality of care metrics for patients presenting with ST-elevation myocardial infarction (STEMI). For patients presenting to the emergency department with chest pain suspicious for an acute coronary syndrome (ACS), the diagnosis can be confirmed by serial EKGs showing ST-T changes and cardiac troponin assays. It is estimated that 60% to 65% of STEMIs occur in patients ≥ 65 years of age and 28% to 33% occur in patients ≥ 75 years of age. Also, as many as 80% of all deaths related to myocardial infarction occur in persons ≥ 65 years of age [13]. The goal for patients with STEMI should be to achieve a door-to-balloon time of ≤ 90 minutes. If immediately available, primary PCI should be performed in patients with STEMI [14].

To the best of our knowledge, the conversion of the WPW pattern into the WPW syndrome in the presence of acute myocardial infarction has not been reported so far. There were animal studies revealing the unmasking of certain pre-excitation pathways in the presence of ischemia [15] but no such human case report or example was available. There have been reports of concomitant myocardial infarction in patients with EKGs positive for pre-excitation [16]. Moreover, there are also case reports of patients with WPW syndrome who required concomitant ablation of the accessory pathway and coronary revascularization procedures [17]. Previously, few reports of the WPW pattern on EKG masking true MI have been documented [18] but none described any case with pre-excited arrhythmia happening in the context of ongoing myocardial ischemia without any such history.

In our case, the diagnosis of acute myocardial infarction was suspected by the presence of chest pain and positive EKG changes with subsequent confirmation by coronary angiography. This case illustrates that primary ST elevation in patients presenting with a WPW pattern is not always a normal variant and may also harbor a pathological and potentially fatal entity. Moreover, pre-excitation with coronary artery disease may not be benign since arrhythmia can readily increase the myocardial oxygen demand and subsequently worsen existing myocardial ischemia (creating a potentially vicious cycle), as was witnessed by the hemodynamic instability requiring immediate cardioversion in this case. This knowledge is clinically important in the current era of reperfusion therapy because a missed diagnosis may not only result in incorrect treatment and potential mortality but also adds to the healthcare cost burden, which is evergrowing globally.

## Conclusions

Our patient was managed adequately and in a timely fashion and is doing well on medical therapy with no reported events on outpatient follow-up evaluations. This raises the potential question of ablation for all manifest accessory pathways, as well as silent pre-excitations, because coronary artery disease is a changing paradigm with more and more cases being reported in younger individuals than previously seen and will require much more work and insight.
